# A Cybersecure P300-Based Brain-to-Computer Interface against Noise-Based and Fake P300 Cyberattacks

**DOI:** 10.3390/s21248280

**Published:** 2021-12-10

**Authors:** Giovanni Mezzina, Valerio F. Annese, Daniela De Venuto

**Affiliations:** 1 Department of Electrical and Information Engineering, Politecnico di Bari, 70125 Bari, Italy; giovanni.mezzina@poliba.it; 2 James Watt School of Engineering, University of Glasgow, Glasgow G12 8LT, UK; Valerio.Annese@glasgow.ac.uk

**Keywords:** P300, brain-to-computer interface, BCI, EEG, machine learning, classification, cybersecurity, hacking, brainjacking

## Abstract

In a progressively interconnected world where the Internet of Things (IoT), ubiquitous computing, and artificial intelligence are leading to groundbreaking technology, cybersecurity remains an underdeveloped aspect. This is particularly alarming for brain-to-computer interfaces (BCIs), where hackers can threaten the user’s physical and psychological safety. In fact, standard algorithms currently employed in BCI systems are inadequate to deal with cyberattacks. In this paper, we propose a solution to improve the cybersecurity of BCI systems. As a case study, we focus on P300-based BCI systems using support vector machine (SVM) algorithms and EEG data. First, we verified that SVM algorithms are incapable of identifying hacking by simulating a set of cyberattacks using fake P300 signals and noise-based attacks. This was achieved by comparing the performance of several models when validated using real and hacked P300 datasets. Then, we implemented our solution to improve the cybersecurity of the system. The proposed solution is based on an EEG channel mixing approach to identify anomalies in the transmission channel due to hacking. Our study demonstrates that the proposed architecture can successfully identify 99.996% of simulated cyberattacks, implementing a dedicated counteraction that preserves most of BCI functions.

## 1. Introduction

A brain-to-computer interface (BCI) is a direct communication channel between the user’s neural activity and electronic devices [1]. Typically, BCIs are based on the recognition of a neural pattern during a specific mental task [1]. The neural activity can be recorded with both invasive and non-invasive equipment. Electroencephalography (EEG) is the most commonly used non-invasive technique in BCI systems [2]. Machine learning and classification algorithms are then used to analyze the neural signals, recognize the target neural pattern, and interpret the user’s intention.

BCIs are growing progressively popular, as they are considered one of the most promising assistive technologies. As such, in the next decade, the global BCI market is expected to increase at a compound annual growth rate (CAGR) of 13.9%, from $1.5 million in 2020 to $5.5 million in 2030 [3]. To date, a variety of neural interfaces have been developed to aid people who have severe neuromuscular disorders [4], including brain-driven spellers [5], cars [1,6], wheelchairs [7], drones [8], and rehabilitative platforms [9]. 

As BCI technology approaches the market, concerns about cybersecurity have been raised [10]. With BCI hacking episodes, we refer to the possibility that a third party may read a patient’s brain states or take control of the interface without the patient’s or healthcare provider’s consent. Currently, the eventuality of hacking is more plausible than ever considering that recent BCI solutions have internet connections [11]. Taking control of a BCI can potentially lead to taking control of the user’s intentions and actions. This raises the terrifying possibility of breaking into systems that control human cognition, feeling, and action and raises ethical concerns pertaining to privacy and physical or psychological harm [12]. The scenario is even more alarming after confirming that most of the current BCI solutions are seriously flawed due to the lack of control against hacking events. BCI uses well-established algorithms and neural patterns, which therefore are hackable. For instance, P300—arguably the most common neural pattern in BCIs—is easily reproducible, as it is widely understood and characterized in the literature. Despite the vulnerability of the current BCI frameworks, no definitive cybersecure solution has been established.

In this paper, we address the cybersecurity challenge by designing, implementing, and validating a novel architecture to monitor EEG headset and BCI framework communication channels. As a proof of concept, we focus on a P300-based interface trained using support vector machine (SVM) algorithms, but the proposed method is versatile and can be applied to other classifiers for BCI. In our study, we initially demonstrated that standard SVM-based P300 BCI interfaces are unable to discriminate real EEG data from synthetic signals. For this aim, we simulated cyberattacks using fake P300 signals. The testing dataset with real P300 data is composed of 7200 trial recordings from 5 different healthy subjects [13]. The testing dataset with fake P300 signals is composed of 2000 trials. Fake P300 signals were synthetically generated using modulated pink noise with a median filter or standard noise-based attacks. Thereafter, we designed and implemented a brain hacking recognizer (BHR), which uses an EEG channel mixing approach to identify anomalies in the transmission channel due to hacking events. We tested the BHR using both real and fake P300 datasets. We demonstrate that the BHR was able to identify 99.996% of attempted cyberattacks. 

## 2. Related Works

Multiple independent scientific studies have demonstrated that BCIs are hackable [10,14,15], regardless of the hardware (e.g., EEG, ECOG, implants [10]), neural pattern (e.g., P300, movement-related potentials), and application (e.g., speller, wheelchair) [14]. In fact, most machine learning algorithms, including SVMs, decision trees, deep belief networks, artificial neural networks, random forest, and naïve Bayes, are vulnerable to cyber threats [15]. In the specific case of P300 classifiers, cyberattacks can cause a relative reduction in the area under the curve (AUC) of up to 74%, depending on the hacker’s knowledge of the user’s data [14]. 

Several solutions, summarized in Table 1, have been proposed to improve the security of a neural interface [16,17,18,19,20,21,22,23,24,25,26,27,28,29,30]. The proposed methods typically use one or more solutions to supervise the BCI session [17,18], authenticate the user [19,20,21,22,23], encrypt the data [24,25,26,27], and ultimately detect cyberattacks [28,29,30].

Supervised BCI systems identify cyberattacks by means of additional sensors and artificial intelligence monitoring the user’s choices. For instance, the use of cameras [17], sensors, and actuators [23] in BCI navigation systems can prevent decisions that are dangerous or the result of hacking. However, additional hardware usually increases the cost and the complexity of the BCI system. 

Authentication-based solutions establish a cybersecure communication channel between the user/sensors and the BCI framework. Authentication can involve the use of additional hardware, including user-specific radio frequency identification (RFID) technology [24], near-field or directional communication [28], and cameras for facial recognition [27]. Brainprint biometric authentication systems with no additional hardware using user-specific signatures in the EEG data have also been developed [20,21]. Nevertheless, the effect of P300 hacking events on authentication systems based on brain signatures is still debatable. 

Encryption techniques protect the communication between the sensors and the BCI framework, arguably the weakest point in BCI systems. Encryption methods for BCI applications include the use of an anonymizer [29], unconventional tensor-based data representation [30], standard encryption algorithms [18], and randomization [19]. Cyberattack identification software has also been developed to identify threats in a timely manner. The dedicated identification software uses the user-specific EEG data [29,30] and profile [20] to identify operational anomalies using artificial intelligence. However, software-based encryption and cyberattack identification solutions typically use complex unconventional algorithms and require high consumption of computational resources. 

These barriers represent a gap to be bridged for implementing cybersecure BCI frameworks in a real-life scenario. As such, we propose a novel approach that uses a low-resource, reproducible, and versatile encryption method together with minimal hardware modifications and therefore is suitable for real-life applications. 

## 3. Simulated Cyberattacks

**Figure 1:**
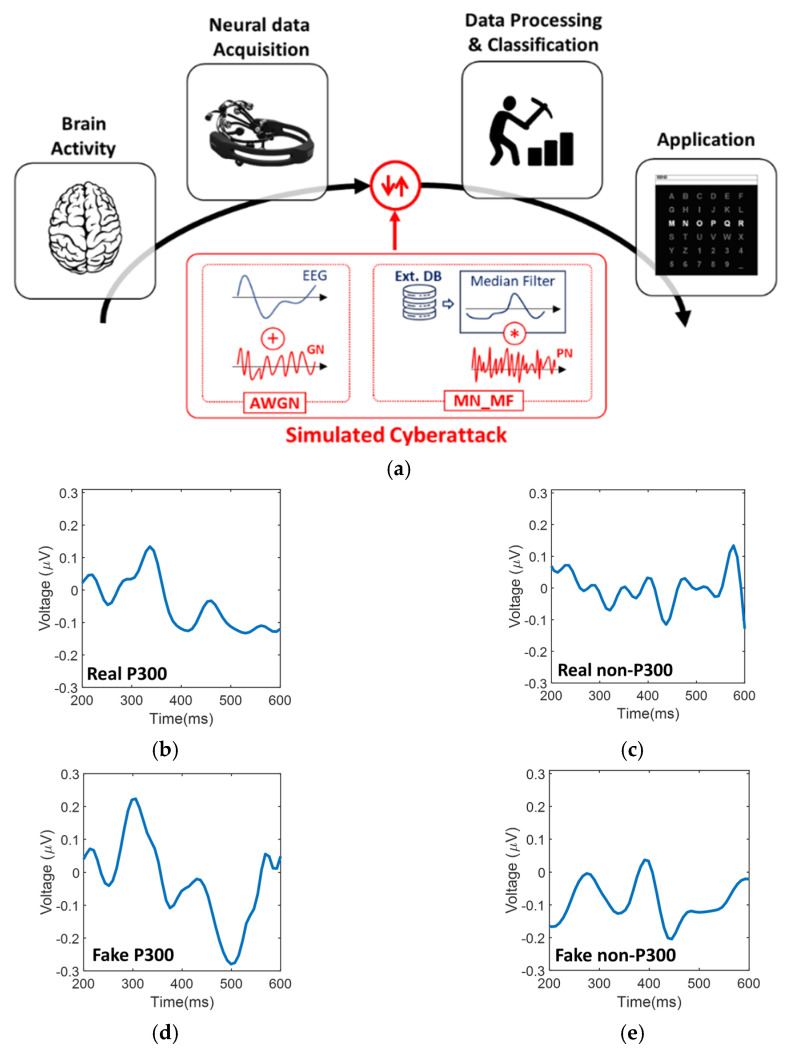
(**a**) BCI processing chain from acquisition to application with simulated cyberattacks on raw data between the neural acquisition and transmission and the BCI framework. (**b**) Example of real P300 and (**c**) real non-P300 signals (subject 1, channel P4, averaged over 200 acquisitions). (**d**) Example of fake P300 and (**e**) fake non-P300 signals (MF3,001, averaged over 200 signals).

Our study focuses on SVMs, which are widely used for BCI implementation. Five different SVM-based BCI models were trained using real P300 data. Then, the models were tested using real P300 data to quantify the performance of the algorithms in identifying P300 for BCI applications. Thereafter, several cyberattacks were simulated. We focused on the specific hacking profile shown in Figure 1, where the attacker hacks the transmission step preceding data processing and classification. We assume that the attackers have knowledge of the hardware (EEG headset), the wireless communication system, and the BCI framework. The attacker also has an external database of EEG signals (“Ext. DB” in Figure 1a) collected with the same settings and equipment. In the simulated cyberattacks, the real P300 dataset was modified by introducing fake signals. The introduction of fake signals was used to demonstrate that SVM-based models fail to recognize that the signal is not legitimate. The weakness of the SVM models was quantified by comparing relevant metrics calculated with real and fake signals. 

### 3.1. Materials and Methods 

**Training dataset (real P300).** A public EEG dataset was used in this work [13]. The training dataset is composed of data from 5 different men who are non-smokers and aged 21–41. None of the participants had any prior BCI experience or a history of neurological disorders. The dataset was produced using the standard 6 × 6 Donchin and Farewell P300 Speller Matrix. The training dataset includes 240 P300 (Figure 1b) and 1200 non-P300 trials (Figure 1c). Data were recorded using the EMOTIV EPOC + EEG wireless headset with 14 channels and a 128 Hz sampling frequency [31]. Data were filtered using two notch filters (50 Hz and 60 Hz) and a bandpass filter (0.2–45 Hz). Additional information on the training dataset can be found in [13].

EEG recordings were pre-processed before training the models according to best practices in the P300 recognition application [32,33,34,35]. Pre-processing was identical regardless of the SVM model to be trained. First, the EEG stream was time windowed between 50 ms and 600 ms after each stimulation onset due to the intrinsic nature of P300 patterns. Indeed, P300 deflection typically occurs in a range from 250 ms to 500 ms, even in the presence of neurocognitive disease [34]. The resulting trials were low-pass filtered with a zero-phase 8th-order Butterworth filter with a cutoff frequency of 15 Hz. This interval was determined to be one of the most discriminative according to a batch of 27 works collected at the 5th Graz BCI Conference [33]. The filtered signals were detrended through a global average approach [33]. Next, the trials were downsampled by a factor of 4 through an average-based technique, achieving 32 Sa/s, reducing the random variations in EEG signals. The last step consisted of a zero-mean and unity standard deviation normalization [32,33,34,35]. The resulting data subset constitutes the features used as input for training the SVM models.

**Testing dataset (real P300).** The testing set is composed of EEG recordings from the same database as above [13]. The testing dataset includes 240 P300 and 1200 non-P300 trials for each of the five participants. Data were acquired using the same equipment and settings as in the training dataset. The pre-processing and feature extraction steps were also replicated from the training dataset.

**Hacked datasets (fake signals).** For each subject, 400 out of 1440 real trials were substituted with fake signals. Real trials substituted with fake ones were randomly selected using the Mersenne Twister 19937 method (seed: 4) [36]. Therefore, fake signals constituted 27.8% of the dataset, mimicking hacking attempts during the normal use of the BCI. Fake P300 (Figure 1d) and non-P300 (Figure 1e) signals were synthetically generated via MATLAB using 5 different methods. As such, five hacked datasets for each of the five participants were generated. The methods used to generate fake signals were:

(i) Addition of white Gaussian noise with SNR = 20 dB (AWGN 20);
(ii) Addition of white Gaussian noise with SNR = 40 dB (AWGN 40);
(iii) Modulated noise with 3rd-order median filter and low-amplitude pink noise (MF3, 001);
(iv) Modulated noise with 9th-order median filter and low-amplitude pink noise (MF9, 001);
(v) Modulated noise with 9th-order median filter and high-amplitude pink noise (MF9, 05).

Methods (i) and (ii) implement a standard noise-based attack with the main objective of altering the acquired EEG signal information content. These methods use a noise model known as additive white Gaussian noise (AGWN). The technique adds Gaussian noise (GN in Figure 1) to the selected EEG signal. By manipulating the signal-to-noise ratio (SNR), it is possible to create various noise scenarios. Specifically, we implemented AWGN by deriving variance from SNR and measured EEG signal power.

Methods (iii)–(v) implement a technique to reproduce synthetic EEG trials containing P300 features in both the time and spectral domains. These methods consider the rejection parameters typically used in the winsorizing process to identify and prevent abnormal voltage amplitudes due to physiological and non-physiological artifacts. However, winsorizing can also be used as a discriminative procedure in noise-based attacks and must be addressed in a fake P300 generation context. The proposed method is schematized in Pseudocode 1.

Real P300 and non-P300 signals were acquired using the same settings and equipment (line 1 of Pseudocode 1). Data were acquired from subjects other than the one under attack that is unknown to the attacker. Next, P300 and non-P300 data from each channel were averaged, resulting in two bidimensional matrices: mP3 for P300 trials and mNP3 for non-P300 ones (lines 3, 4). To emphasize only those features common to all P300/non-P300 trials over all analyzed subjects, a channel-specific median filter was applied (lines 5, 6).

Different nth-order unidimensional median filters were investigated. Median filtering is widely applied in image processing. The median filter replaces the center value in a selected window with the median value of all of the points within the window:

(1) $$y\left(m\right)=\mathrm{median}\left(x\left(m-\frac{n-1}{2}:\;m+\frac{n-1}{2}\right)\right)$$

where y and **x** are the filter output and input signals, respectively, m is the considered sample index, and n is the filter order.

To generalize the attack, invalidating all of the main performance metrics of the BCI, both targets and non-targets are independently cyberattacked. A random selection of the median signal to be used for the attack generation is carried out at line 7 of Pseudocode 1.

**Pseudocode 1. Routine for fake P300 generation based on modulated noise with a median filter.**

1. **load** P300_Trials, NotP300_Trials

2. # Generate attack

3.     mP3      ←  mean(P300_trials)

4.     mNP3      ←  mean(NotP300_trials)

5.     MF_mP3     ←  medianfilter(mP3)

6.     MF_mNP3     ←  medianfilter(mNP3)

7.     atck       ←  random_select (MF_mP3, MF_mNP3)

8. **for **(channel):

9.     EEG_lims       ←  **extract** EEG(channel) streaming limits

10.     atck_lims      ←  **extract** atck(channel) streaming limits

11.     k          ←  adapt_factor(EEG_lims, atck_lims)

12.     pn          ←  generatePinkNoise

13.     → **generate**  MN_MF fake P300(channel)

Next, for each channel, the attacker simulator extracts the EEG amplitude limits from the signal streaming and the same parameters from the median-filtered waveforms. This process allows the system to generate a correction factor for the waveform to prevent its amplitude from approaching the winsorizing limits, preventing amplitude-based attack identification (line 11).

Finally, the attack waveform is generated as:

(2) $$MN\_MF=((\alpha_{1}\cdot pn)*\;MF\_sig)\cdot k$$

where MN_MF is a generated attack (P300 or non-P300), and $\alpha_{1}$ is a hyperparameter that manages the amplitude of the locally generated pink noise vector pn. MF_sig represents the median-filtered signal regardless of whether it is P300 or non-P300, while k represents the correction function for protection against winsorizing. The dataset composition is summarized in Table 2.

**SVM Training.** For each subject, five different SVM-based BCI models were trained using the respective training set. The implemented SVMs have (i) linear (L), (ii) quadratic (Q), (iii) cubic (C), (iv) medium Gaussian (MG), and (v) coarse Gaussian (CG) kernels. The models were trained using the MATLAB Classification Learner tool by MathWorks with a k-fold cross-validation (k = 5) approach. Table 2 summarizes the datasets and methods used. For each subject and each model, we calculated accuracy, precision, recall, and F1 (defined in Table 2). For each of the above metrics, a comparative parameter named “cyberattack effect” was introduced in this study. It consists of the difference between the same metrics calculated with real and fake P300 data.

### 3.2. Results of the Simulated Cyberattack

**Figure 2:**
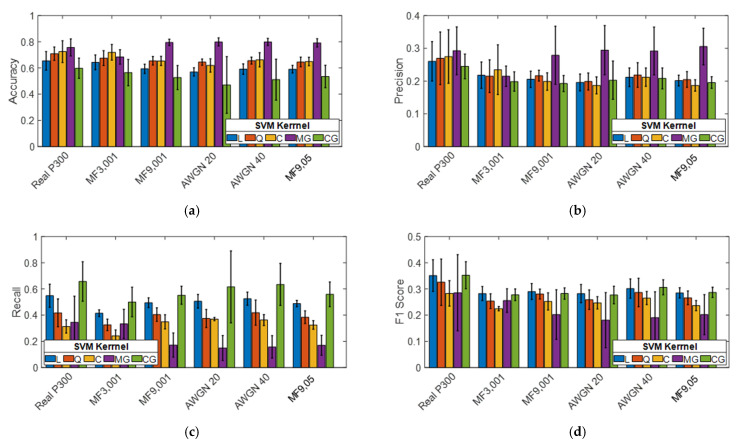
Test of the SVM models using the real P300 dataset (real P300) vs. hacked P300 datasets. Performance metrics are (**a**) accuracy, (**b**) precision, (**c**) recall, and (**d**) F1 score. Data are represented as averages and standard deviations over the five subjects.

Each trained model was individually tested using all of the testing datasets in Table 2. The results of the simulated cyberattack are summarized in Figure 2. Data are presented as averages and standard deviations over the five subjects. Subject-specific data are reported in Tables S1–S5 of the Supplementary Materials of this work. Figure 2 shows that the presence of cyberattacks generated using (MF9, 001), (AWGN20), (AWGN40), and (MF9, 05) leads to a reduction in precision and F1-score parameters compared to the real P300 dataset regardless of the used SVM model. On average, all of the parameters are affected by the introduction of fake signals.

**Figure 3:**
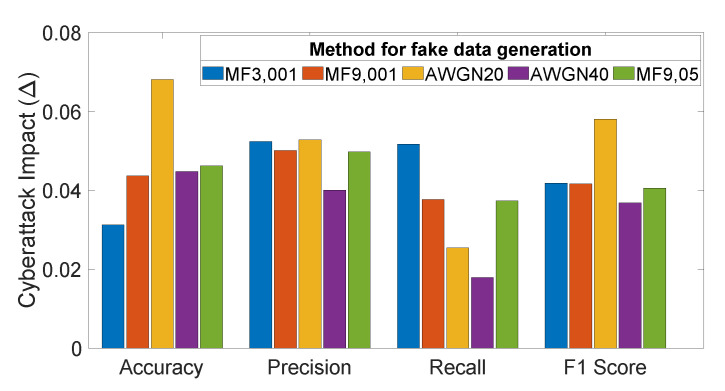
Metric reduction versus BCI performance metrics for each cyberattack method.

Figure 3 shows the cyberattack impact, as defined in Table 2. Averaging parameter values over the five subjects and the five SVM kernels results in a reduction in the accuracy parameter, which ranges between 3% (MF3, 001) and 7% (AWGN 20). Precision is reduced by about 5% with all of the cyberattacks except for AWGN 40, which reduces the precision by 4%. Recall shows a reduction between 2% (AWGN 40) and 5% (MF3, 001). The F1-score decreases by 4% in most cases, except for AWGN 20, which achieves a 6% decrease. Considering the overall cyberattack impact, as shown in Figure 3, the attack using fake signals generated with (MF3, 001) is demonstrated to be the most effective hacking method. This method simultaneously reduces all parameters relative to those calculated on the real P300 dataset, regardless of the used SVM model.

The simulated cyberattacks primarily show that standard classification algorithms are incapable of identifying cyberattacks, consequently losing their overall accuracy in discriminating P300 trials because of wrong classification outcomes. The fake P300 data eluded all of the SVM models used in this study. This performance is intolerable in a real-life application. These simulation results are in line with findings from [10,14,15], raising serious concerns about the security of most BCIs.

## 4. Architecture of the Brain Hacking Recognizer (BHR) for the Cybersecure BCI

**Figure 4:**
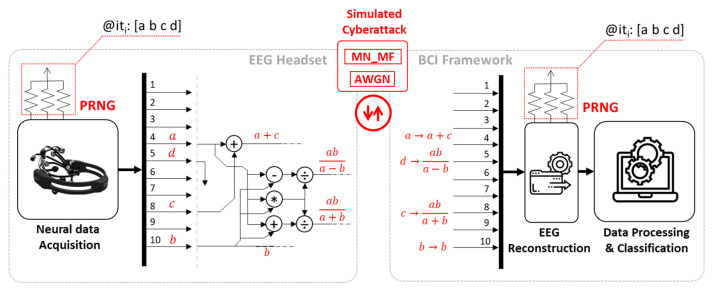
Architecture of a 10-channel BHR.

The proposed cybersecure architecture is based on encrypted multi-channel communication between the EEG headset and the BCI framework. We designed and implemented a brain hacking recognizer (BHR) architecture that supervises the communication between the EEG headset and the BCI framework. The BHR aims to (i) detect communication anomalies and potential cyberattacks, (ii) supply the BCI framework with possible actions to inhibit the attack, and (iii) lead the BCI to a secure status. Figure 4 depicts the proposed architecture.

### 4.1. Brain Hacking Recognizer

The working principle of the BHR is based on a pseudo-random number generator (PRNG). A PRNG consists of a set of pull-up resistors identifying a hardware-specific random seed replicated on both the EEG headset and the BCI framework [37]. As such, the PRNG supplies known random numbers to the EEG headset and BCI framework. The random numbers are deterministic if the seed and iteration numbers are known (as in the presented application).

At a generic ith iteration, the PRNG on the EEG headset side generates four indexes (a, b, c, and d in Figure 4). As per the demonstrative BHR overview on 10 channels in Figure 4, these indexes are used to randomly select four specific EEG channels (e.g., in the ith iteration, index a corresponds to EEG channel 4). Data on the selected channels are mixed using algebraic operations in Figure 4, with channel b transmitted unaltered. The new channel composition is buffered and transmitted to the BCI framework.

Thereafter, the BCI framework receives the data. The BCI framework, having the same PRNG setting and iteration number, is aware of the four indexes a, b, c, and d. Data on the channel indexed as b represent a reference pattern. The same signal can also be estimated using the remaining channels a, c, and d using appropriate inverse operations, as shown in Figure 4. Let us use $\hat{b}$ to denote the estimation of the signal on channel b using only data from channels a, c, and d. The BHR estimates $\hat{b}$ using the following inverse operations:

(8) $$\frac{\mathrm{ab}}{a+b}=x\;\;\;\;\;\frac{\mathrm{ab}}{a-b}=y\;$$

(9) $$\frac{1}{b}+\frac{1}{a}=\frac{1}{x}\;\;\;\;\;\frac{1}{b}-\frac{1}{a}=\frac{1}{y}$$

Let us define:

(10) $$\frac{1}{b}=e\;\;\;\;\;\;\;\frac{1}{a}=f$$

Considering the sum among the equations composing the system defined by Equations (9) and (10) results in:

(11) $$e=\frac{\left(\frac{1}{c}+\frac{1}{d}\right)}{2}\;\to \hat{b}$$

where $\hat{b}$ is the estimation of the signal on the channel indexed as b.

If $\hat{b}$ coincides with data received on channel b, the BHR marks the transmission as legitimate and proceeds with the estimation of a and the reconstruction of signal on channel c, feeding the data processing and classification block discussed in Section 2. In detail, if the transmission is attack-free, the reconstruction returns, ideally, $\hat{b}=b$ because channels indexed as c and d are unaltered. In this case, the signal on the channel indexed as a can be estimated from $\hat{b}$ according to:

(12) $$f=\frac{1}{x}-e\;\to \hat{a}$$

Obtaining the estimation $\hat{a}$, it is also possible to derive the signal on channel c on the EEG headset side.

If $\hat{b}$ does not match data received on channel b, the BHR marks the transmission as “compromised” and plans a counteraction to inhibit the attack. In the presence of a cyberattack, we assume that the attacker is unaware of the seed (a, b, c, and d) and iteration number. Therefore, even if an attack based on the P300 trial with physiological characteristics is generated, the attacker will modify the channels, making channel b reconstruction impossible on the BCI framework side. Moreover, even if the attacker is aware of the channel mixing methodology, it has a very low probability of finding the right disposition among the available ones. A detailed analysis of this probability is provided in Section 3.2.

In any case, an erroneous attack trial would lead to $\hat{b}\neq b$. In this case, a cyberattack inhibition step follows the BHR alert (i.e., BCI under attack). It is beyond the scope of this work to define the best counterattack, as this is application-dependent. However, as a proof of concept, let us consider a P300 speller. The unsafe situation in a P300 speller is the presence of observations erroneously considered to be positive (i.e., false positive). This situation would lead to incorrect word spelling. In this case, false negatives do not affect the proper functioning of the system. For cyberattacks on P300-based spellers, we, therefore, recommend forcing the BCI to classify the corrupted trial as non-P300, blocking its operation with an attack warning for the user. The concept can be easily expanded to a wheelchair or car that drives through a BCI [38].

### 4.2. Results of the BHR Rejection Capabilities

To demonstrate that the BHR is effective in rejecting cyberattacks, a testbench composed of a total of 144 million attack trials was tested. The testbench was realized on the basis of 100,000 sessions. Each session consisted of 1440 attacks (i.e., fake signals), which represent the whole dataset. In this study, we assumed that the hacker is aware of the presence of the BHR and has knowledge of the PRNG hardware/algorithm, but the seed and iteration number are unknown. This represents the worst-case scenario.

**Figure 5:**
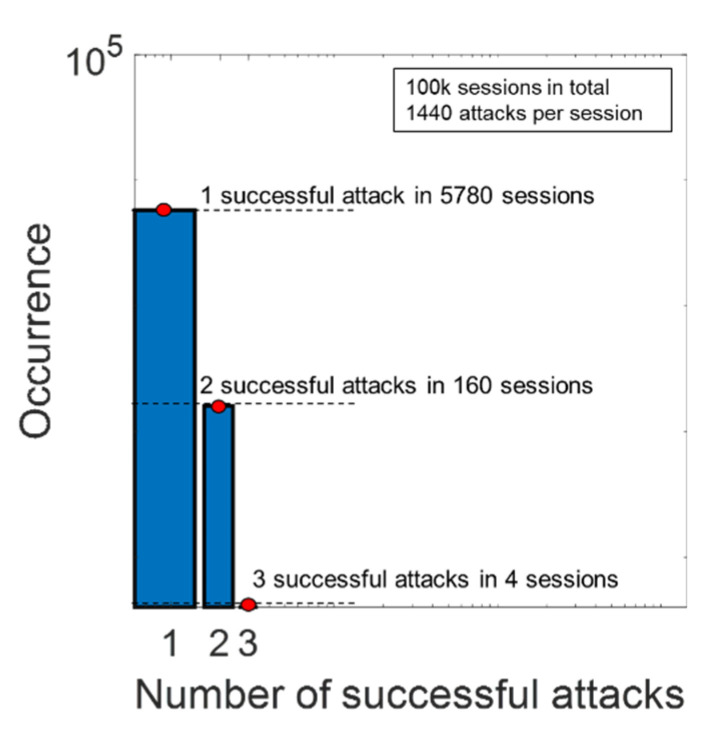
Frequency of successfully hacked trials of 100,000 sessions composed of 1440 attacks (whole dataset).

Figure 5 depicts the occurrence of successfully hacked trials per session. The results show that in 5780 sessions out of 100,000 (5.78%), a single trial out of 1440 available was successfully hacked. In the same context, 160/100,000 sessions (0.16%) recorded the successful corruption of two trials. Similarly, 4/100,000 sessions (0.004%) presented three affected trials. Overall, 6112 attacks out of 144 million trials (0.0042%) overcame the BHR protection system. Therefore, BHR was successful in rejecting 99.996% of the cyberattack trials regardless of the hacking type (e.g., noise-based or physiological-like attack).

To provide a complete overview of the proposed brainjacking recognition method, the computational time of the overall processing chain on the BCI framework receiver side was assessed. The test was conducted on a laptop PC with Intel Core i5-10210U and 8 GB RAM. Starting from the stimulus onset, the whole processing chain on the receiver side required a total of 612.7 ms. Specifically, the EEG data buffer filling for the trial definition required 600 ms (97.9% of the total time), while 11.58 ± 0.38 ms (2% of the total time) was required in the classification stage. The introduction of the BHR increased the processing time by 0.826 ms ± 0.69 ms (0.1% of the total time) on average. Therefore, the adoption of the implemented architecture has a negligible impact on the computational time of the system, allowing it to readily detect and inhibit a cyberattack.

### 4.3. Results of the Simulated Cyberattack with BHR

The five previously trained SVM models were supplied with the proposed BHR. Each new BCI was tested on the hacked P300 datasets reported in Table 2. The nature of the BHR working principle leads the system to react in the same way regardless of the hacking type. Indeed, according to the inhibition procedure, the BHR forces the BCI to label the trial as non-P300 as soon as it detects discrepancies between signals on specific channels. This happens for any considered hacking type, resulting in a unique BCI-dependent outcome.

**Figure 6:**
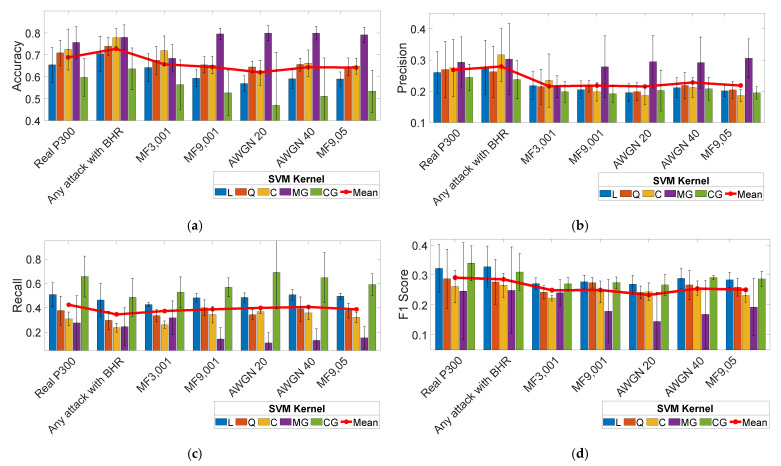
Test of the SVM models using the real P300 dataset (real P300) vs. hacked datasets. Performance metrics are (**a**) accuracy, (**b**) precision, (**c**) recall, and (**d**) F1-score. Data are presented as averages and standard deviations over the five subjects. The black line with markers reports the average value of the metric calculated on the five SVM kernels.

Figure 6 shows the performance metric behavior when the real P300 dataset (reference) or the hacked P300 datasets are used to test the BCIs with and without the BHR. The first and last five blocks of histograms are repurposed from Figure 3. The second block of histograms reports the mean value and standard deviation of the specific parameter computed on the five subjects with different SVM kernels when BCIs are supplied with the BHR. The mean value over the five SVM models is also highlighted with a dotted red line in Figure 6.

**Figure 7:**
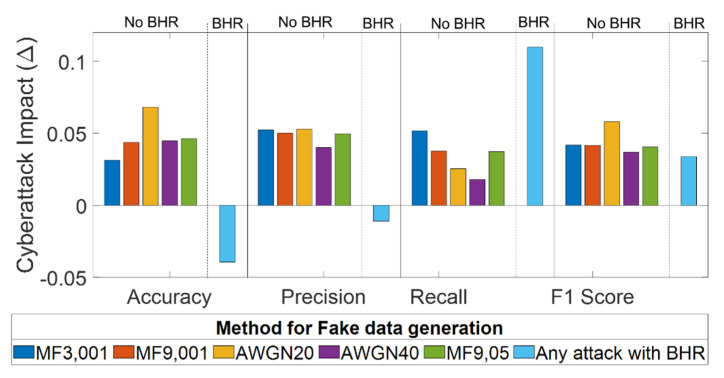
Metric reduction versus BCI performance metrics for each cyberattack method.

The results in Figure 7 show that, overall, the BCIs supplied with the proposed BHR improve their accuracy, precision, and F1-score parameters at the expense of the recall metric. Indeed, as per Equation (5), the increase in FN, with a consequent reduction in TP, due to the attack on P300 trials (constrained with the label non-P300 by the inhibition procedure) hardly affected the BCI recall. Figure 7 illustrates the impact on BCI performance through the cyberattack impact parameter, as defined in Table 2. The results demonstrate that accuracy is improved relative to the real P300 dataset reference by about 4% and by 7.1% for the less effective attack (i.e., the MF3, 001). Similarly, the precision increases by 1.1% relative to the reference and 5.1% for the less effective attack (i.e., AWGN 40). The F1-score is still reduced relative to the reference performance by 3.3%; however, the BHR introduction improves the F1-score by 0.3% if compared with the AWGN 40 dataset result (less effective attack in terms of F1-score). Conversely, the recall parameter shows a large decrease of 10.9% considering the reference value and 5.8% relative to the most effective attack source (i.e., MF3, 001). Subject-specific data are reported in Tables S6–S10 of the Supplementary Materials of this work.

### 4.4. Discussion

The approach proposed in this work applies to a specific scenario where (i) the hacker attacks the communication channel between sensors and the BCI framework; (ii) the hacker has knowledge of the hardware, (iii) wireless communication system, and (iv) BCI framework; (v) the attacker uses EEG signals from other subjects other than the user under attack; and (vi) fake P300 signals are synthetically created using noise-based and median filter techniques. Arguably, the scenario proposed in this work is one of the most plausible. However, there are many other scenarios where the hacker operates differently or has different degrees of knowledge. For instance, the attacker might be able to access user-specific data or might have direct access to the BCI framework. The assessment of the BHR performance in different hacking scenarios is beyond the scope of this work and will be investigated in future works. Nevertheless, since the working principle of the BHR is based on a low-resource pseudo-random algebraic combination of the data to find anomalies in communication before the BCI framework operation, the nature and the provenience of data (i.e., P300 from the same subject, different subjects, synthetic or simply noise) are irrelevant. The minimum requirement for this architecture to work is to have at least four synchronized communication channels from the sensors to the BCI framework. Although we used EEG data to demonstrate the architecture, the data type is irrelevant to the proper functioning of the cybersecure framework. Therefore, we envisage that the proposed architecture can be applied to other multi-channel systems for measuring neural potentials, including electrocorticography (ECOG) [39] and local field potentials (LFP).

Furthermore, the BHR reconstructs data on the BCI framework end before any classification or machine learning is performed. As such, the features of the neural pattern and the algorithm used for its classification do not affect the BHR operation. Although we demonstrated the proposed solution for a P300 SVM-based BCI, the BHR is modular and can be applied to recognize hacking attempts on BCI systems based on other patterns (e.g., as movement or pre-movement brain potentials) and algorithms (e.g., neural networks, decision tree, random forest) [40].

## 5. Conclusions

Cybersecurity is an aspect that has received far less attention when designing and implementing a BCI. In this work, a novel architecture to improve cybersecurity in BCI systems is presented. The working principle of the architecture is based on the transmission of a linear combination of EEG data on randomly selected and dynamically changing communication channels. This approach allows identifying breaches of the wireless communication between the EEG headset and the BCI framework. Wireless communication is indeed the weakest point in current BCI solutions, as it is exposed to external cyberattacks.

Our study shows that with no security features, current BCI systems are exposed to cyber threats. By using simulated cyberattacks, we demonstrate that SVM models, which are very popular in BCIs, are incapable of discriminating real EEG data from fake signals. After implementing the proposed cybersecurity solution, the BCI framework was able to identify 99.996% of simulated cyberattacks. As such, the BCI system can therefore implement routines to both inhibit further attacks and ensure the safe functioning of the user interface according to the specific application.

The architecture proposed in this study is versatile and can be easily implemented on a variety of BCI models. We believe that our study can also raise awareness of the risk of using exposed BCI systems so that designers will dedicate special attention to this aspect of the interface.

## Figures and Tables

**Table 1:** State-of-the-art cybersecure frameworks.

Method	Solution	Application	Ref.
Supervision	Navigation unit (camera, laser odometry)	P300-based navigation system	[17]
	Navigation unit (wheel encoders, proximity sensors, gyro sensor, and an RGBD sensor)	P300-driven wheelchair	[23]
Authentication	RFID technology	Bidirectional BCI	[24]
	Near-field communication	User framework	[28]
	Facial recognition	IoT framework	[27]
	Brainprint biometric authentication	P300 speller	[25]
		BCI framework	[26]
Encryption	BCI anonymizer	BCI Framework	[29]
	Tensor-based data representation	EEG data	[30]
	Chaotic encryption	EEG data	[18]
	Randomization	BCI Framework	[19]
Cyberattack identification software	User-specific action profile	User framework	[20]
	User-specific EEG data	EEG data	[21]
		P300 BCI	[22]

**Table 2:** Datasets used in this study and metrics of interest.

**DATASETS**					
**Dataset**	**Data Type**	**Method**		**Description**	
Training	Real data	EEG acquisition		5 subjects, 1440 trials per subject	
Testing	Real data	EEG acquisition		5 subjects, 1440 trials per subject	
	Hacked data	EEG + AWGN 20		5 subjects, 1040 real trials per subject 400 fake trials per subject	
		EEG + AWGN 40			
		EEG + MF3,001			
		EEG + MF9,001			
		EEG + MF9,05			
**METRICS**					
**Parameter**			**Relation ^1^**		
Accuracy (A)			$A=\frac{\mathrm{TP}\;+\;\mathrm{TN}}{\mathrm{TP}\;+\;\mathrm{TN}\;+\;\mathrm{FP}\;+\;\mathrm{FN}}$		(3)
Precision or positive predicted value (PPV)			$\mathrm{PPV}=\frac{\mathrm{TP}}{\mathrm{TP}\;+\;\mathrm{FP}}$		(4)
Recall or true positive rate (TPR)			$\mathrm{TPR}=\frac{\mathrm{TP}}{\mathrm{TP}\;+\;\mathrm{FN}}$		(5)
F1 score (F1)			$F1=2\frac{\mathrm{PPV}\;\cdot \mathrm{TPR}}{\mathrm{PPV}\;+\;\mathrm{TPR}}$		(6)
Cyberattack impact (Δ)			$\Delta =V_{\mathrm{real}\;}-V_{\mathrm{fake}\;}$		(7)

^1^ TP: true positives; TN: true negatives; FP: false positives; FN: false negatives; V_real_: generic metric calculated with real P300 dataset; V_fake_: same metric calculated with fake P300 dataset.

## Data Availability

In this study, we used a publicly available EEG dataset [13]. The dataset has been modified to simulate cyberattacks, as described in Section 2.

## Supplementary Materials

The following are available online at www.mdpi.com/article/10.3390/s21248280/s1, Tables S1–S5: Test of the SVM models using the real P300 dataset (real P300) vs. hacked P300 datasets for Subject *number of subject from 1 to 5*. Tables S6–S10: Test of the SVM models using the real P300 dataset (real P300) vs. hacked P300 datasets for Subject *number of subject from 1 to 5* when BCI is supplied with the proposed BHR.
