# Supplementary material for: A Cybersecure P300-Based Brain-to-Computer Interface against Noise-Based and Fake P300 Cyberattacks
Source: Sensors (Basel). 2021 Dec 10;21(24):8280. doi: 10.3390/s21248280 (PMC8709057; doi:10.3390/s21248280)
Supplement: Supplementary file 1 [file sensors-21-08280-s001.zip › sensors-1488183-supplementary.pdf]

sensors

MDPI

Supplementary Materials

A Cybersecure P300-based Brain-to-Computer Interface Against Noise-Based and Fake P300 Cyberattacks

Giovanni Mezzina [1*†], Valerio F. Annese [2†] and Daniela De Venuto [1,*]

1 Department of Electrical and Information Engineering, Politecnico di Bari, Bari, Italy
2 James Watt School of Engineering, University of Glasgow, United Kingdom
* Correspondence: {giovanni.mezzina, daniela.devenuto}@poliba.it
† Both authors contributed equally to this work.

Sup1. Results of Simulated Cyberattack on Subjects

With reference to Sec. 2.2 , each trained Support Vector Machine (SVM) model was individually tested using all the testing datasets proposed in Table 1 of the main text. Tables S1 – S5 summarizes the effect of the cyberattacks generated using (MF9, 001), (AWGN20), (AWGN40), and (MF9, 05) on the single analyzed subject according to the dataset [22].

Table S1. Test of the SVM models using the real P300 dataset (Real P300) vs. hacked P300 datasets for Subject 1.

Accuracy					
**SVM Kernel**	**L**	**Q**	**C**	**MG**	**CG**
**Real P300**	0.729861	0.790278	0.809722	0.741667	0.676389
**MF3,001**	0.745833	0.774306	0.815972	0.713889	0.708333
**MF9,001**	0.647222	0.683333	0.691667	0.775	0.649306
**AWGN 20**	0.607639	0.659028	0.6375	0.798611	0.773611
**AWGN 40**	0.630556	0.690278	0.702083	0.786806	0.660417
**MF9,05**	0.622917	0.672222	0.672917	0.756944	0.638194
**Precision**					
**SVM Kernel**	**L**	**Q**	**C**	**MG**	**CG**
**Real P300**	0.347648	0.408824	0.412371	0.346512	0.291513
**MF3,001**	0.292763	0.309417	0.380952	0.262431	0.254098
**MF9,001**	0.24717	0.241627	0.232984	0.309091	0.2334
**AWGN 20**	0.233169	0.243354	0.196121	0.368421	0.319328
**AWGN 40**	0.248276	0.277056	0.246649	0.320856	0.263757
**MF9,05**	0.211429	0.228972	0.206107	0.25	0.212679
**F1-score**					
**SVM Kernel**	**L**	**Q**	**C**	**MG**	**CG**
**Real P300**	0.466392	0.47931	0.368664	0.444776	0.404092
**MF3,001**	0.327206	0.298056	0.231884	0.315615	0.306931
**MF9,001**	0.34026	0.306991	0.286174	0.295652	0.31479
**AWGN 20**	0.334511	0.326475	0.258523	0.325581	0.317992
**AWGN 40**	0.35122	0.364672	0.300163	0.28103	0.362451
**MF9,05**	0.290196	0.293413	0.255924	0.23913	0.285322
**Recall**					
**SVM Kernel**	**L**	**Q**	**C**	**MG**	**CG**
**Real P300**	0.708333	0.579167	0.333333	0.620833	0.658333
**MF3,001**	0.370833	0.2875	0.166667	0.395833	0.3875
**MF9,001**	0.545833	0.420833	0.370833	0.283333	0.483333
**AWGN 20**	0.591667	0.495833	0.379167	0.291667	0.316667
**AWGN 40**	0.6	0.533333	0.383333	0.25	0.579167
**MF9,05**	0.4625	0.408333	0.3375	0.229167	0.433333

Table S2. Test of the SVM models using the real P300 dataset (Real P300) vs. hacked P300 datasets for Subject 2.

**Accuracy**					
**SVM Kernel**	**L**	**Q**	**C**	**MG**	**CG**
**Real P300**	0.627777778	0.686111111	0.740277778	0.775	0.552083333
**MF3,001**	0.624305556	0.655555556	0.700694444	0.73125	0.582638889
**MF9,001**	0.580555556	0.613888889	0.649305556	0.815277778	0.505555556
**AWGN 20**	0.559027778	0.598611111	0.615277778	0.802083333	0.327083333
**AWGN 40**	0.566666667	0.625694444	0.660416667	0.805555556	0.459722222
**MF9,05**	0.590277778	0.605555556	0.648611111	0.806944444	0.528472222
**Precision**					
**SVM Kernel**	**L**	**Q**	**C**	**MG**	**CG**
**Real P300**	0.241258741	0.25462963	0.272108844	0.317391304	0.217573222
**MF3,001**	0.208897485	0.198113208	0.202492212	0.218390805	0.192504259
**MF9,001**	0.206451613	0.213768116	0.21987315	0.383928571	0.195090439
**AWGN 20**	0.180936995	0.175	0.174273859	0.258064516	0.17309417
**AWGN 40**	0.204615385	0.215238095	0.220224719	0.303921569	0.191513761
**MF9,05**	0.20735786	0.202898551	0.197727273	0.330357143	0.210803689
**F1-score**					
**SVM Kernel**	**L**	**Q**	**C**	**MG**	**CG**
**Real P300**	0.339901478	0.327380952	0.299625468	0.310638298	0.326018809
**MF3,001**	0.285336856	0.253012048	0.231729055	0.22754491	0.273276904
**MF9,001**	0.297674419	0.297979798	0.291725105	0.244318182	0.297830375
**AWGN 20**	0.260768335	0.239473684	0.232686981	0.144144144	0.284870849
**AWGN 40**	0.298876404	0.295424837	0.286131387	0.18128655	0.300359712
**MF9,05**	0.295942721	0.282828283	0.255882353	0.210227273	0.32032032
**Recall**					
**SVM Kernel**	**L**	**Q**	**C**	**MG**	**CG**
**Real P300**	0.575	0.458333333	0.333333333	0.304166667	0.65
**MF3,001**	0.45	0.35	0.270833333	0.2375	0.470833333
**MF9,001**	0.533333333	0.491666667	0.433333333	0.179166667	0.629166667
**AWGN 20**	0.466666667	0.379166667	0.35	0.1	0.804166667
**AWGN 40**	0.554166667	0.470833333	0.408333333	0.129166667	0.695833333
**MF9,05**	0.516666667	0.466666667	0.3625	0.154166667	0.666666667

Table S3. Test of the SVM models using the real P300 dataset (Real P300) vs. hacked P300 datasets for Subject 3.

**Accuracy**					
**SVM Kernel**	**L**	**Q**	**C**	**MG**	**CG**
**Real P300**	0.652777778	0.656944444	0.733333333	0.639583333	0.484027778
**MF3,001**	0.622222222	0.6125	0.700694444	0.576388889	0.489583333
**MF9,001**	0.578472222	0.684027778	0.684722222	0.759722222	0.533333333
**AWGN 20**	0.566666667	0.663888889	0.634722222	0.741666667	0.670138889
**AWGN 40**	0.592361111	0.625694444	0.678472222	0.752083333	0.566666667
**MF9,05**	0.586805556	0.669444444	0.678472222	0.751388889	0.554861111
**Precision**					
**SVM Kernel**	**L**	**Q**	**C**	**MG**	**CG**
**Real P300**	0.236842105	0.223913043	0.233333333	0.214723926	0.189122373
**MF3,001**	0.200787402	0.188235294	0.190938511	0.200647249	0.194066749
**MF9,001**	0.189509306	0.222222222	0.188953488	0.259090909	0.180473373
**AWGN 20**	0.192307692	0.20952381	0.189130435	0.2	0.178082192
**AWGN 40**	0.204429302	0.205128205	0.208877285	0.227906977	0.2
**MF9,05**	0.199661591	0.210784314	0.180515759	0.245689655	0.198496241

F1-score					
**SVM Kernel**	**L**	**Q**	**C**	**MG**	**CG**
**Real P300**	0.31880109	0.294285714	0.247058824	0.288065844	0.291706387
**MF3,001**	0.272727273	0.256	0.214936248	0.289044289	0.299332698
**MF9,001**	0.269554753	0.274322169	0.22260274	0.247826087	0.266375546
**AWGN 20**	0.277777778	0.266666667	0.248571429	0.191304348	0.214876033
**AWGN 40**	0.290205562	0.278447122	0.25682183	0.215384615	0.290909091
**MF9,05**	0.283995187	0.265432099	0.213921902	0.241525424	0.291712707
**Recall**					
**SVM Kernel**	**L**	**Q**	**C**	**MG**	**CG**
**Real P300**	0.4875	0.429166667	0.2625	0.4375	0.6375
**MF3,001**	0.425	0.4	0.245833333	0.516666667	0.654166667
**MF9,001**	0.466666667	0.358333333	0.270833333	0.2375	0.508333333
**AWGN 20**	0.5	0.366666667	0.3625	0.183333333	0.270833333
**AWGN 40**	0.5	0.433333333	0.333333333	0.204166667	0.533333333
**MF9,05**	0.491666667	0.358333333	0.2625	0.2375	0.55

Table S4. Test of the SVM models using the real P300 dataset (Real P300) vs. hacked P300 datasets for Subject 4.

Accuracy					
**SVM Kernel**	**L**	**Q**	**C**	**MG**	**CG**
**Real P300**	0.536805556	0.665277778	0.567361111	0.829166667	0.588194444
**MF3,001**	0.572916667	0.6375	0.633333333	0.691666667	0.420833333
**MF9,001**	0.544444444	0.615277778	0.59197787	0.820138889	0.372222222
**AWGN 20**	0.518055556	0.650694444	0.526388889	0.831944444	0.196527778
**AWGN 40**	0.530555556	0.6625	0.559722222	0.83125	0.230555556
**MF9,05**	0.538888889	0.597916667	0.5875	0.827083333	0.379166667
**Precision**					
**SVM Kernel**	**L**	**Q**	**C**	**MG**	**CG**
**Real P300**	0.172043011	0.169398907	0.16344464	0.2	0.276299113
**MF3,001**	0.172774869	0.164285714	0.166666667	0.166666667	0.164785553
**MF9,001**	0.17192429	0.190944882	0.156	0.12	0.158436214
**AWGN 20**	0.159159159	0.172069825	0.146964856	0.25	0.16751269
**AWGN 40**	0.165644172	0.162087912	0.161512027	0.2	0.167687596
**MF9,05**	0.170807453	0.160320641	0.155642023	0.304347826	0.163580247
**F1-score**					
**SVM Kernel**	**L**	**Q**	**C**	**MG**	**CG**
**Real P300**	0.251402918	0.204620462	0.229913473	0.016	0.423712342
**MF3,001**	0.243542435	0.209090909	0.214285714	0.186813187	0.259325044
**MF9,001**	0.249427918	0.259358289	0.209115282	0.022641509	0.254125413
**AWGN 20**	0.233995585	0.215288612	0.212471132	0.008196721	0.285361334
**AWGN 40**	0.242152466	0.195364238	0.228710462	0.008163265	0.283311772
**MF9,05**	0.248868778	0.216508796	0.212201592	0.053231939	0.262376238
**Recall**					
**SVM Kernel**	**L**	**Q**	**C**	**MG**	**CG**
**Real P300**	0.466666667	0.258333333	0.3875	0.008333333	0.908333333
**MF3,001**	0.4125	0.2875	0.3	0.2125	0.608333333
**MF9,001**	0.454166667	0.404166667	0.317073171	0.0125	0.641666667
**AWGN 20**	0.441666667	0.2875	0.383333333	0.004166667	0.9625
**AWGN 40**	0.45	0.245833333	0.391666667	0.004166667	0.9125
**MF9,05**	0.458333333	0.333333333	0.333333333	0.029166667	0.6625

Table S5. Test of the SVM models using the real P300 dataset (Real P300) vs. hacked P300 datasets for Subject 5.

**Accuracy**					
**SVM Kernel**	**L**	**Q**	**C**	**MG**	**CG**
**Real P300**	0.725	0.746527778	0.774305556	0.797916667	0.688194444
**MF3,001**	0.647916667	0.697916667	0.745138889	0.709722222	0.621527778
**MF9,001**	0.618055556	0.678472222	0.649305556	0.80625	0.575
**AWGN 20**	0.596527778	0.652777778	0.681944444	0.81875	0.384027778
**AWGN 40**	0.636111111	0.675694444	0.710416667	0.818055556	0.638194444
**MF9,05**	0.6125	0.684027778	0.659027778	0.815972222	0.572916667
**Precision**					
**SVM Kernel**	**L**	**Q**	**C**	**MG**	**CG**
**Real P300**	0.304020101	0.2909699	0.292682927	0.384615385	0.24940048
**MF3,001**	0.214132762	0.214076246	0.232067511	0.225308642	0.186858316
**MF9,001**	0.211895911	0.213367609	0.195402299	0.321100917	0.195081967
**AWGN 20**	0.213445378	0.193396226	0.226130653	0.396039604	0.174874372
**AWGN 40**	0.235074627	0.232941176	0.222570533	0.406779661	0.217303823
**MF9,05**	0.218085106	0.220779221	0.193154034	0.396694215	0.191103789
**F1-score**					
**SVM Kernel**	**L**	**Q**	**C**	**MG**	**CG**
**Real P300**	0.379310345	0.322820037	0.269662921	0.368763557	0.316590563
**MF3,001**	0.282885431	0.251290878	0.230607966	0.258865248	0.250343879
**MF9,001**	0.293059126	0.26391097	0.251851852	0.200573066	0.28
**AWGN 20**	0.304191617	0.246987952	0.282131661	0.234604106	0.281781377
**AWGN 40**	0.324742268	0.297744361	0.254025045	0.268156425	0.293080054
**MF9,05**	0.305970149	0.272	0.243451464	0.265927978	0.27390791
**Recall**					
**SVM Kernel**	**L**	**Q**	**C**	**MG**	**CG**
**Real P300**	0.504166667	0.3625	0.25	0.354166667	0.433333333
**MF3,001**	0.416666667	0.304166667	0.229166667	0.304166667	0.379166667
**MF9,001**	0.475	0.345833333	0.354166667	0.145833333	0.495833333
**AWGN 20**	0.529166667	0.341666667	0.375	0.166666667	0.725
**AWGN 40**	0.525	0.4125	0.295833333	0.2	0.45
**MF9,05**	0.5125	0.354166667	0.329166667	0.2	0.483333333

Sup2. Results of Simulated Cyberattack on Subjects – BCI with BHR

With reference to Sec. 3.3 , the five trained SVM models were supplied with the proposed Brain Hacking Recognizer (BHR) and were tested on the hacked P300 datasets reported in Table 1 of the main text. Tables S6 – S10 summarizes the effect of the cyberattacks generated using (MF9, 001), (AWGN20), (AWGN40), and (MF9, 05) on the single analyzed subject according to the dataset [22] when the model is supplied with the BHR.

Table S6. Test of the SVM models using the real P300 dataset (Real P300) vs. hacked P300 datasets for Subject 1 when BCI is supplied with the proposed BHR.

**Accuracy**					
**SVM Kernel**	**L**	**Q**	**C**	**MG**	**CG**
**Real P300**	0.729861111	0.790277778	0.809722222	0.741666667	0.676388889
**Any Attack with BHR**	0.754166667	0.786805556	0.818055556	0.756944444	0.704166667
**Precision**					
**SVM Kernel**	**L**	**Q**	**C**	**MG**	**CG**
**Real P300**	0.347648262	0.408823529	0.412371134	0.346511628	0.291512915
**Any Attack with BHR**	0.328313253	0.365461847	0.426666667	0.322580645	0.263959391
**F1-score**					
**SVM Kernel**	**L**	**Q**	**C**	**MG**	**CG**
**Real P300**	0.466392318	0.479310345	0.368663594	0.444776119	0.404092072
**Any Attack with BHR**	0.381118881	0.372188139	0.328205128	0.363636364	0.32807571
**Recall**					
**SVM Kernel**	**L**	**Q**	**C**	**MG**	**CG**
**Real P300**	0.708333333	0.579166667	0.333333333	0.620833333	0.658333333
**Any Attack with BHR**	0.708333333	0.379166667	0.266666667	0.416666667	0.433333333

Table S7. Test of the SVM models using the real P300 dataset (Real P300) vs. hacked P300 datasets for Subject 2 when BCI is supplied with the proposed BHR.

**Accuracy**					
**SVM Kernel**	**L**	**Q**	**C**	**MG**	**CG**
**Real P300**	0.627777778	0.686111111	0.740277778	0.775	0.552083333
**Any Attack with BHR**	0.683333333	0.720138889	0.754861111	0.790972222	0.582638889
**Precision**					
**SVM Kernel**	**L**	**Q**	**C**	**MG**	**CG**
**Real P300**	0.241258741	0.25462963	0.272108844	0.317391304	0.217573222
**Any Attack with BHR**	0.232673267	0.255255255	0.255411255	0.303225806	0.179396092
**F1-score**					
**SVM Kernel**	**L**	**Q**	**C**	**MG**	**CG**
**Real P300**	0.339901478	0.327380952	0.299625468	0.310638298	0.326018809
**Any Attack with BHR**	0.291925466	0.296684119	0.250530786	0.237974684	0.251556663
**Recall**					
**SVM Kernel**	**L**	**Q**	**C**	**MG**	**CG**
**Real P300**	0.575	0.458333333	0.333333333	0.304166667	0.65
**Any Attack with BHR**	0.391666667	0.354166667	0.245833333	0.195833333	0.420833333

Table S8. Test of the SVM models using the real P300 dataset (Real P300) vs. hacked P300 datasets for Subject 3 when BCI is supplied with the proposed BHR.

**Accuracy**					
**SVM Kernel**	**L**	**Q**	**C**	**MG**	**CG**
**Real P300**	0.652777778	0.656944	0.733333	0.639583	0.484028
**Any Attack with BHR**	0.711111111	0.702083	0.793056	0.69375	0.524306
**Precision**					
**SVM Kernel**	**L**	**Q**	**C**	**MG**	**CG**
**Real P300**	0.236842105	0.223913	0.233333	0.214724	0.189122
**Any Attack with BHR**	0.267195767	0.201893	0.335227	0.208696	0.168405
**F1-score**					
**SVM Kernel**	**L**	**Q**	**C**	**MG**	**CG**
**Real P300**	0.31880109	0.294286	0.247059	0.288066	0.291706
**Any Attack with BHR**	0.326860841	0.229803	0.283654	0.246154	0.248079
**Recall**					
**SVM Kernel**	**L**	**Q**	**C**	**MG**	**CG**
**Real P300**	0.4875	0.429167	0.2625	0.4375	0.6375
**Any Attack with BHR**	0.420833333	0.266667	0.245833	0.3	0.470833

Table S9. Test of the SVM models using the real P300 dataset (Real P300) vs. hacked P300 datasets for Subject 4 when BCI is supplied with the proposed BHR.

**Accuracy**					
**SVM Kernel**	**L**	**Q**	**C**	**MG**	**CG**
**Real P300**	0.536805556	0.665277778	0.567361111	0.829166667	0.588194444
**Any Attack with BHR**	0.583333333	0.708333333	0.717361111	0.829166667	0.611111111
**Precision**					
**SVM Kernel**	**L**	**Q**	**C**	**MG**	**CG**
**Real P300**	0.172043011	0.169398907	0.16344464	0.2	0.276299113
**Any Attack with BHR**	0.167896679	0.166666667	0.213058419	0.2	0.265395894
**F1-score**					
**SVM Kernel**	**L**	**Q**	**C**	**MG**	**CG**
**Real P300**	0.251402918	0.204620462	0.229913473	0.016	0.423712342
**Any Attack with BHR**	0.232736573	0.176470588	0.233521657	0.016	0.392624729
**Recall**					
**SVM Kernel**	**L**	**Q**	**C**	**MG**	**CG**
**Real P300**	0.466666667	0.258333333	0.3875	0.008333333	0.908333333
**Any Attack with BHR**	0.379166667	0.1875	0.258333333	0.008333333	0.754166667

Table S10. Test of the SVM models using the real P300 dataset (Real P300) vs. hacked P300 datasets for Subject 5 when BCI is supplied with the proposed BHR.

**Accuracy**					
**SVM Kernel**	**L**	**Q**	**C**	**MG**	**CG**
**Real P300**	0.725	0.746527778	0.774305556	0.797916667	0.688194444
**Any Attack with BHR**	0.794444444	0.779166667	0.809027778	0.829166667	0.761111111
**Precision**					
**SVM Kernel**	**L**	**Q**	**C**	**MG**	**CG**
**Real P300**	0.304020101	0.2909699	0.292682927	0.384615385	0.24940048
**Any Attack with BHR**	0.391472868	0.322727273	0.352941176	0.480769231	0.310218978
**F1-score**					
**SVM Kernel**	**L**	**Q**	**C**	**MG**	**CG**
**Real P300**	0.379310345	0.322820037	0.269662921	0.368763557	0.316590563
**Any Attack with BHR**	0.40562249	0.308695652	0.233983287	0.378787879	0.3307393
**Recall**					
**SVM Kernel**	**L**	**Q**	**C**	**MG**	**CG**
**Real P300**	0.504166667	0.3625	0.25	0.354166667	0.433333333
**Any Attack with BHR**	0.420833333	0.295833333	0.175	0.3125	0.354166667
